# Evaluation of the Healing Effect of Ointments Based on Bee Products on Cutaneous Lesions in Wistar Rats

**DOI:** 10.3390/ph14111146

**Published:** 2021-11-11

**Authors:** Calin Vasile Andritoiu, Cristina Lungu, Maricel Danu, Bianca Ivanescu, Corina Elena Andriescu, Laurian Vlase, Corneliu Havarneanu, Camelia Elena Iurciuc (Tincu), Marcel Popa

**Affiliations:** 1 Apitherapy Medical Center, Balaneşti, 217036 Gorj, Romania; dr_calin_andritoiu@yahoo.com; 2 Nutrition and Dietetics Specialization, Faculty of Pharmacy, Vasile Goldis Western University of Arad, 310025 Arad, Romania; 3 Department of Pharmaceutical Botany, Faculty of Pharmacy, Grigore T. Popa University of Medicine and Pharmacy, 700115 Iasi, Romania; 4 Petru Poni Institute of Macromolecular Chemistry, 700487 Iasi, Romania; mdanu@tuiasi.ro; 5 Department of Natural and Synthetic Polymers, Cristofor Simionescu Faculty of Chemical Engineering and Environmental Protection, Gheorghe Asachi Technical University of Iasi, 700050 Iasi, Romania; camelia_tincu83@yahoo.com (C.E.I.); marpopa2001@yahoo.fr (M.P.); 6 Department of Pathology, Sf. Spiridon Emergency County Hospital, 700111 Iaşi, Romania; andriescu_corina@yahoo.co.uk; 7 Department of Pharmaceutical Technology and Biopharmacy, Faculty of Pharmacy, Iuliu Hatieganu University of Medicine and Pharmacy, 400012 Cluj-Napoca, Romania; laurian.vlase@yahoo.com; 8 Faculty of Psychology and Education Sciences, Alexandru Ioan Cuza University, 700554 Iasi, Romania; hcornel@uaic.ro; 9 Department of Pharmaceutical Technology, Faculty of Pharmacy, Grigore T. Popa University of Medicine and Pharmacy, 700115 Iasi, Romania; 10 Academy of Romanian Scientists, 050094 Bucharest, Romania

**Keywords:** bee products, honey, propolis, apilarnil, wound healing, rheological characterization

## Abstract

The wound-healing capacity of ointments based on bee products was investigated in vivo on three experimental models of incision, excision and heat burn. For this purpose, four ointments were prepared with propolis, honey, apilarnil (drone brood homogenate) and a mixture of these three apitherapy products. The ointments were applied topically for 21 days. Clinical and macroscopic evaluation was performed throughout the experiment, with the recording of the re-epithelialization period and determination of the wound contraction rate on days 6 and 9. The histopathological examination was performed on days 1, 3, 12 and 21 of the treatment. The topical formulations were also characterized from a rheological point of view in order to verify their stability. HPLC analysis of propolis revealed the presence of phenolic compounds, particularly ferulic acid and p-coumaric which were found in high amounts. All ointments had beneficial effects on wound contraction and the re-epithelialization period, but the most significant result, both macroscopically and especially in terms of histological architecture, was presented by the ointment that contains all three apitherapy products, due to their synergistic effect.

## 1. Introduction

Bee products such as honey, pollen, propolis, bee bread, royal jelly and drone larvae have been used around the world since ancient times as sources of food, medicine and cosmetics, based on their diverse chemical composition including numerous bioactive compounds.

Honey has a long history of use as a therapeutic agent in wound healing not only due to its cellular and tissue regenerating action but also because of its antiseptic, anti-inflammatory and analgesic properties. Sugars (fructose, glucose, maltose) are the major components of honey, but it also includes proteins, amino acids, enzymes, vitamins, minerals, organic acids, flavonoids and phenolic acids. The composition and antioxidant activity of honey depend on the botanical origin, pedoclimatic conditions and production process [1].

Propolis is a mixture of resinous and gummy substances, waxes, essential oils and pollen used by bees for protecting the hives from invaders and microbial pathogens, for heat insulation and for reinforcing the hives. The chemical composition depends primarily on the botanical origin, and the main bioactive compounds of therapeutic interest are flavonoids, benzoic acids, cinnamic acids, caffeoylquinic acids, terpenoids and amino acids. With over 200 active compounds in its composition, propolis has antimicrobial, anti-inflammatory, free radical scavenging, immunomodulatory and regenerative properties, being a natural mixture of antioxidants that act synergistically [2].

A less known bee product is drone brood homogenate or apilarnil, which is obtained from the whole composition of 7-day-old drone larvae comb cells by trituration, homogenization, filtration and lyophilization. These operations are needed for ensuring the quality of the product which degrades rapidly after harvest and loses its biological properties. Drones are male bees that emerge from unfertilized eggs, and their sole purpose is to mate with the queen [3]. Apilarnil has a high content of proteins and amino acids (glutamic acid, leucine, aspartic acid, proline, lysine, valine, alanine, taurine and phosphoserine), as well as carbohydrates (glucose, fructose, trehalose, maltose). In addition, it is a good source of lipids (triglycerides, saturated fatty acids, mono- and polyunsaturated fatty acids and phytosterols), vitamins (choline, thiamine, pantothenic acid, riboflavin, pyridoxine, retinol, β-carotene, calciferol and α-tocopherol) and minerals (mainly sodium, potassium, calcium, magnesium and phosphorus). Drone brood also contains hormones (estradiol, prolactin, progesterone and testosterone) and the highest amount of antioxidant polyphenols among bee products [4]. Apilarnil has hypolipidemic, antianemic, immunostimulatory, hepatoprotective, androgenic, neuroprotective and adaptogenic properties and improves fertility and libido [5]. Being a concentrate of vitalizing, regenerating and therapeutic substances, similar to royal jelly, drone brood is an excellent candidate for the treatment of burns.

Continuing the research direction on obtaining nontoxic formulas for the treatment of skin wounds, such as products with natural polymers [6] or vegetal extracts [7], this study aims to evaluate the healing effect of some bee products in incision, excision and burn lesions.

## 2. Results

### 2.1. HPLC Analysis of Propolis and Honey Samples

Propolis owes its numerous biological activities mainly to polyphenols, such as flavonoids, phenolic acids and their esters. Therefore, the hydroalcoholic propolis extract was chemically analyzed by three analytical methods to highlight the bioactive compounds that could be responsible for the wound healing action of propolis ointment, and the concentrations of identified compounds are included in Table 1.

**Figure 1:**
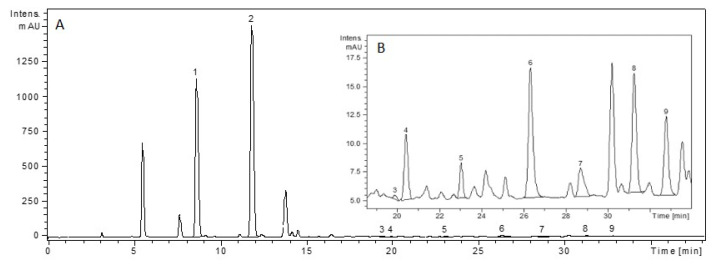
HPLC chromatogram of hydroalcoholic propolis extract. (**A**) Global view (1—p-coumaric acid, 2—ferulic acid). (**B**) Close-up of signal intensity of time range 19–34 min (3—isoquercitrin, 4—rutoside, 5—quercitrin, 6—quercetin, 7—luteolin, 8—kaempferol, 9—apigenol).

The main phenolic compounds in the hydroalcoholic propolis extract were ferulic acid and p-coumaric acid, both found in high concentrations, as shown in Figure 1.

Gentisic acid, hyperoside and myricetin were identified in the hydroalcoholic propolis extract by MS-MS detection but were not quantified, because the UV signal was under the limit of quantification or interferences/peak overlapping from other compounds did not allow the quantitative determination (Figure S1). Fisetin, patuletin, sinapic acid, caftaric acid, eupatorin, eupatilin and casticin were not found in the hydroalcoholic extract of propolis. Moderate amounts of flavones (apigenin, luteolin), flavonols (quercetin, kaempferol) and quercetin glycosides (rutoside, quercitrin, isoquercitrin) were determined in the hydroalcoholic propolis extract. Among the six methoxylated flavonols analyzed, acacetin, jaceosidin and hispidulin were found in small quantities.

The oily extract of propolis and the honey sample were analyzed by the HPLC-UV-MS method with 18 standards. In the propolis oily extract, three phenolic acids were quantified, namely caffeic acid (19.13 µg/mL), p-coumaric acid (71.66 µg/mL) and ferulic acid (99.60 µg/mL), while gentisic acid was only identified by MS. In the honey sample, gentisic acid alone was qualitatively determined by MS spectra but was not quantified by UV, due to its low amount (under the limit of quantification). Chromatograms of honey and oily propolis samples with the identified compounds are included in the Supplementary Information file (Figure S2–S4, Table S1).

### 2.2. Rheological Characterization

Both rheological tests (oscillatory and rotational tests) were performed. The reproducibility of the results was verified by performing rheological tests on three samples of each analyzed ointment.

**Figure 2:**
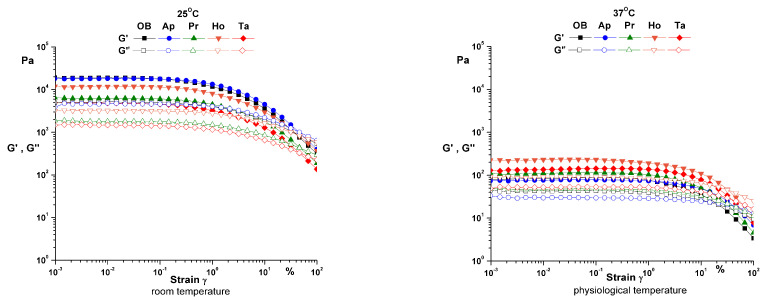
Amplitude sweep for analyzed ointments at both temperatures (25 and 37 °C). OB—ointment base, Ap—ointment base + apilarnil, Pr—ointment base + propolis, Ho—ointment base + honey, Ta—ointment base + mixture of bee products.

The first oscillatory rheological test performed on the samples was the amplitude sweep (Figure 2) to estimate the limit of the linear viscoelastic range and the structural stability of the ointments. The amplitude sweep test was performed at constant angular frequency (ω = 10 rad/s) and constant temperature (25 °C and 37 °C) in the strain range ɣ = 0.001–100%.

**Figure 3:**
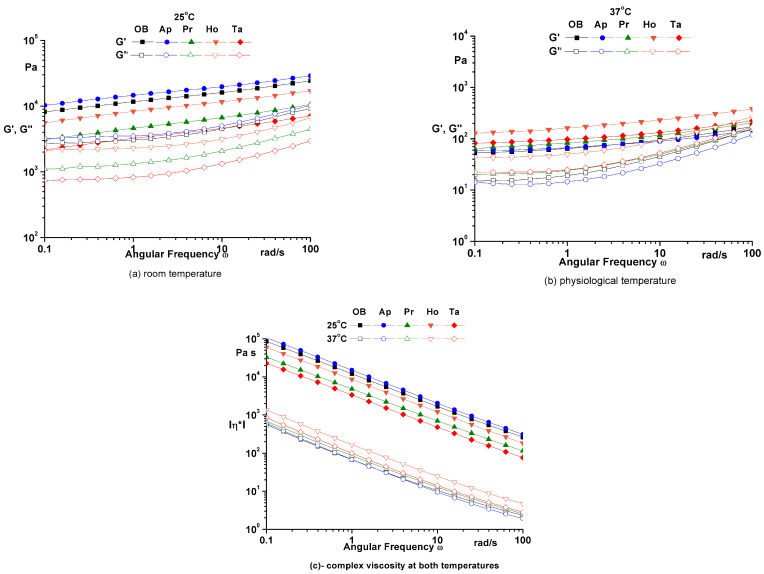
Frequency sweep for analyzed ointments. OB—ointment base, Ap—ointment base + apilarnil, Pr—ointment base + propolis, Ho—ointment base + honey, Ta—ointment base + mixture of bee products.

The frequency sweep was performed at constant amplitude (γ = 0.1%), constant temperature (25 °C and 37 °C) and variable angular frequency (ω = 0.1–100 rad/s). In the frequency sweep, the storage modulus G’, loss modulus G” at 25 °C (Figure 3a) and 37 °C (Figure 3b) and complex viscosity at both temperature (Figure 3c) over the entire frequency range were recorded.

**Figure 4:**
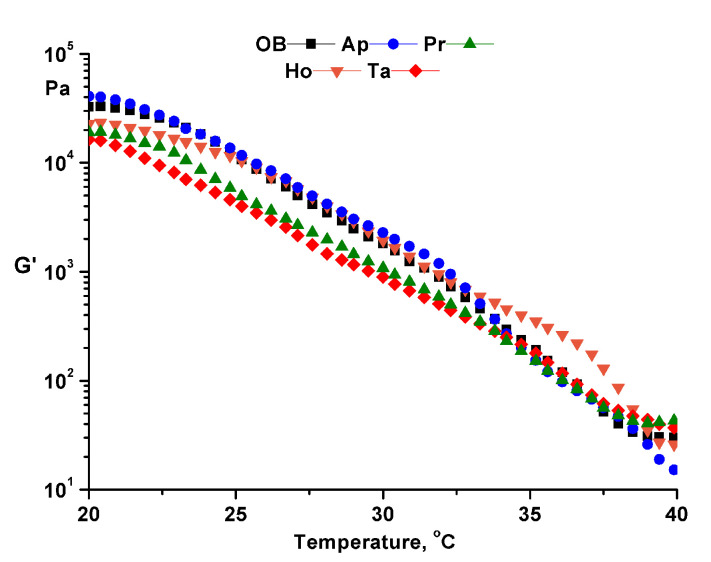
Temperature test for analyzed ointments. OB—ointment base, Ap—ointment base + apilarnil, Pr—ointment base + propolis, Ho—ointment base + honey, Ta—ointment base + mixture of bee products.

Dynamic temperature sweep tests (Figure 4) were performed between 20 and 40 °C with 0.5 °C/min rate at a constant angular frequency of 10 rad/s and a constant strain in the linear viscoelastic region (γ = 0.1%). 

**Figure 5:**
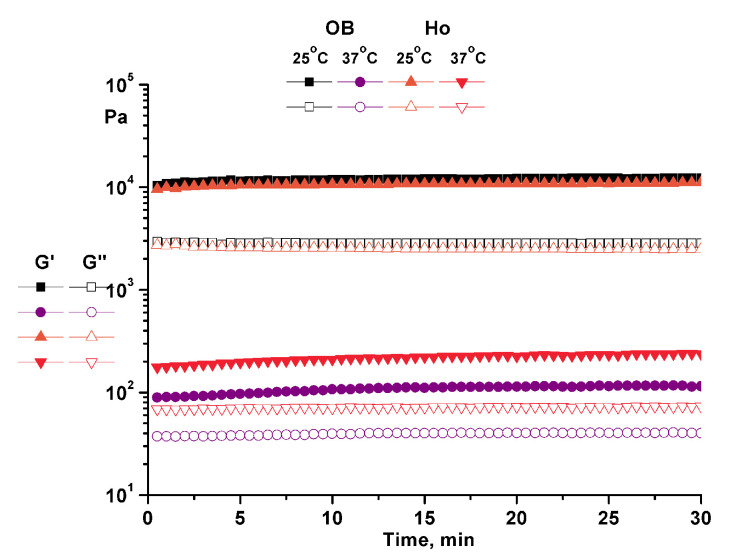
Time test for analyzed ointment. OB—ointment base, Ap—ointment base + apilarnil, Pr—ointment base + propolis, Ho—ointment base + honey, Ta—ointment base + mixture of bee products.

Time test (Figure 5) was performed in a dynamic oscillation (constant strain) mode (strain amplitude 0.1% and angular frequency 10 rad/s). 

**Figure 6:**
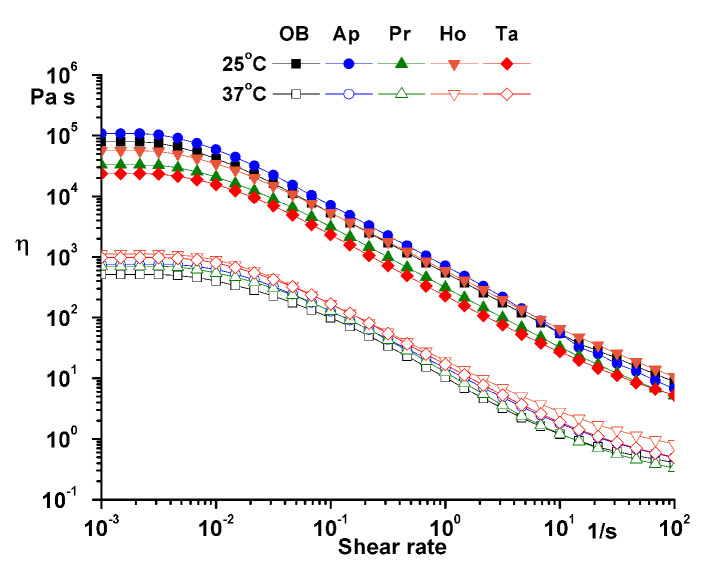
Flow curves for analyzed ointments. OB—ointment base, Ap—ointment base + apilarnil, Pr—ointment base + propolis, Ho—ointment base + honey, Ta—ointment base + mixture of bee products.

Flow curves (Figure 6) were recorded in the 0.001 to 100 s^−1^ domain at constant temperature (25 and 37 °C).

### 2.3. Wound Healing Evaluation Parameters

Clinical and macroscopic evaluation was performed throughout the study, recording the re-epithelialization period; determining the rate of contraction of the lesion (days 6 and 9); and performing the histopathological examination on days 1, 3, 12 and 21 of the treatment.

#### 2.3.1. Period of Re-epithelialization and Wound Contraction Rate

The wound areas were measured on days 0, 6 and 9. The results are presented in Table 2. 

Regarding excision-type lesions, no significant changes were recorded in terms of wound contraction in the first three days of postinjury treatment. Inflammatory phenomena and perilesional edema were observed, with local clinical exacerbation for the thermal burn lesion; in such cases, surgical excision of the affected tissue is recommended. The cell proliferation begins after three days, and a significant reduction in wound area (*p* < 0.001) was obtained on days 6 and 9, with positive results for all treated groups. However, it can be noted that the Pr group displayed the best effects on day 6 of the study, compared to the OB group (WCR = 73.06 ± 1.36% vs. 26.74 ± 2.13%) and especially compared to the NC group (WCR = 5.78 ± 1.86%).

On day 9 of treatment, the results were obviously positive for the Pr group (WCR = 97.97 ± 0.16%), followed by Ta group (WCR = 96.54 ± 0.18%) and Ho group (WCR = 93.65 ± 0.28%). On day 12 of treatment, the re-epithelialization process was completed for the wound excision model, the re-epithelialization area being 1 × 1 mm^2^ (Pr group).

The Ta group indicated favorable effects from day 6 of the study (WCR = 72.50 ± 0.36%) compared to the OB group and especially compared to the NC group. On day 12 of treatment, the re-epithelialization process was completed for the wound excision model (1 × 1.2 mm^2^). 

#### 2.3.2. Histological Examination 

The relevant pathological anatomy results are presented with discussions on the micrographs captured for each of the groups on days 1, 3, 12 and 21 of the study (Table 3, Table 4, Table 5 and Table 6).

The three models, incision, excision and thermal burn, displayed a similar aspect in terms of the numbers of lymphocytes (1–2/high-power field (HPF)) and fibroblasts (2/HPF for excision and 3–4/HPF for incision and thermal burn), with discrete edema in the dermis. The number of capillaries is similar: 1–2/HPF for excision and thermal burn and 2–3/HPF for incision, with slight parietal thickening of the vessels in the hypodermis, accompanied by congestion for the excision-type lesion. Fibrosis is discrete in the superficial dermis with modest accentuation of the basement membrane (excision-type), slightly more accentuated in the incision-type model and severe in the thermal burn. The dermal edema is discrete in all three models. The epidermis contains 1–2 layers in the excision and incision models (Table 3).

For the NC group, the incision displayed an area of necrosis (ulceration), most likely adjacent; the presence of chronic inflammatory infiltrate and a significant abscess in the keratin layer was observed in the hypodermis. The dermis adjacent to the area of necrosis (fistula) indicated inflammatory elements and edema. Additionally, vacuolar degeneration and isolated dehiscence between stratum basale and stratum spinosum were observed. Regarding the excision, a microabscess was observed in the stratum corneum that continued with ulceration (fistula) to the hypodermis; at this, a significant chronic inflammatory infiltrate was noted. The muscle fragment showed severe edema, and a submuscular collection with chronic inflammatory elements was noticed. In excision, acanthosis and vacuolar degeneration were observed, as was slight submuscular necrosis. In the thermal burn, the ulceration of the epidermis was identified with the presence of a rich inflammatory infiltrate and of severe fibrosis in the superficial and deep dermis. The hypodermis presented moderate chronic inflammation and thick-walled vessels. The epidermis presented areas of dyskeratosis, and the hypodermis displayed necrosis of muscle fibers. The abscess in the corneous layer extended into the dermis with an area of necrosis at this level. Chronic periadnexal inflammation was also found in the deep and middle dermis (Table 3).

For the OB group, in incision, we noted a significant inflammatory infiltrate in the hypodermis and muscular interstitium, lymphocytes and edema in the superficial dermis and remaining vessels; in excision, we noted the appearance of myositis and the presence of ulceration, significant inflammatory infiltrate in the hypodermis, perivascular lymphocytes and necrotic–hemorrhagic detritus; in thermal burn, we observed inflammation in the hypodermis and muscle layer. Moreover, additional features were present: perivascular lymphocytes, periadnexial inflammatory infiltrate and aspects of myositis (Table 3).

For the Ho group, similar aspects were found in the three experimental models, with moderate inflammatory infiltrate in the hypodermis; muscle fragment of normal aspect was found in the incision and thermal burn models with mild interstitial inflammatory infiltrate associated with edema. There were few inflammatory elements, 2–3/HPF in the dermis, slight congestion, mild edema in excision and moderate edema in incision. Discrete collagenization was noted in excision and incision; in the thermal burn, more important dermal collagenization of the superficial dermis was noted. Another note refers to the similar number of fibroblasts (2–3/HPF) in excision and incision and 2 fibroblasts/HPF in thermal burn. The epidermis had a normal aspect (Table 3).

For the Pr group, we noticed the following features: In the hypodermis, for the incision-type lesion, there was a moderate chronic inflammatory infiltrate plus a relatively normal muscle fragment, with moderate edema. Submuscularly, there was a small collection of chronic inflammatory elements for the excision-type lesion. Additionally, we observed vessels with a thickened wall in the hypodermis. For the thermal burn-type lesion, pronounced fibrosis was found in the deep dermis, and the hypodermis had moderate chronic inflammation and thick-walled vessels; normal-looking muscle with moderate edema and isolated muscle fibers with slightly different diameters were observed (Table 3).

For the Ap group, in the incision, a fragment of connective tissue with fibroblasts and capillaries was observed. In excision, namely in the hypodermis and muscle fragment, we observed moderate inflammatory infiltrate and interstitial fibrosis that includes many fibroblasts. In thermal burns, we noted moderate or accentuated fibrosis in the superficial and middle dermis. The deep dermis and hypodermis showed connective tissue transformation with connective fibers that penetrated the muscle fibers. Moreover, we noticed a moderate chronic inflammatory infiltrate in the muscle fragment. In the depth of the muscle fragment, mature granulation tissue with fibrosis, few neoformation vessels and inflammatory elements were present (Table 3).

For the Ta group, the following aspects were noted in the superficial dermis: reduced inflammatory elements, 2 lymphocytes/HPF, 2 fibroblasts/HPF in excision, 5 fibroblasts/ HPF in incision, mild edema and few capillaries in excision and incision and slight collagenization in incision and excision. The middle dermis and hypodermis displayed moderate chronic inflammation in the excision and pronounced inflammation in the incision, with severe involvement of the striated muscle fibers and submuscular adipose tissue in the incision (Table 3).

On day 12 of treatment, for the NC group, we recorded the following aspects: giant cells in the incision, dermal collagenization, inflammatory infiltrate and congestion in the hypodermis and dermis. Exocytosis for the incision and important inflammatory infiltrate in the hypodermis for excision were displayed. The burn-type lesion was represented by a rich inflammatory infiltrate in the hypodermis (Table 5).

For the OB group, in the incision-type lesion, we observed keratin-induced foreign-body giant cell, congestion in the hypodermis and in the excision-type lesion, increased collagenization, the appearance of fibrosis, significant congestion and inflammatory infiltrate. In the thermal burn, the periadnexal inflammatory infiltrate noticed on day 3 was still maintained on day 12.

For the Ho group, regarding the incision, at the level of the dermis we highlighted the following: rare lymphocytic elements (2–3/HPF) with discrete edema; few congestive capillaries (1/HPF); mixed inflammatory infiltrate, moderate, in the hypodermis; muscle fragment of normal aspect. At the level of excision, we observed maturing granulation tissue with increased number of vessels, located from the superficial dermis to the hypodermis. For thermal burn, we noted the upper dermis having mature granulation tissue and the middle dermis showing moderate densification with the horizontalization of collagen fibers. The hypodermis presented mild inflammation and associated congestion; muscle fragment was of normal aspect. The epidermis was relatively normal in all three models.

For the Pr group in the incision, it was found that the fistula line was replaced with mature granulation tissue from the epidermis to the hypodermis. Immediately below the epidermis, we remarked a thick fibrotic band. In the hypodermis, mild inflammation and vascular congestion were noted; in excision, mature granular tissue that extends to the hypodermis was noted. Under the epidermis, in the superficial dermis, vascular congestion was observed; near to the hypodermis, chronic inflammatory elements were observed. In the thermal burn, similar to the incision, a band of fibrosis was found under the epidermis. In the middle dermis, a relatively normal aspect with loose connective tissue was displayed. At the level of the hypodermis, minor inflammatory infiltrate and associated vascular congestion were also present.

For the Ap group, the following aspects were recorded: the presence of lymphocytes in the incision, an important dermal collagenization in the excision-type lesion and dermal collagenization in the burn-type lesion.

The superficial dermis for the To group presented similar features in the three models, with 2–3 lymphocytes/HPF, 2–3 fibroblasts/HPF, 1 capillary/HPF, slight edema and mild or absent fibrosis (collagenization) in the incision and excision models and moderate fibrosis in the thermal burn, with the horizontalization and homogenization of the collagen fibers. The epidermis was rectilinear, with 2-3 layers, in the three models. The excision showed a thickened epidermis under which a mature granulation tissue was formed. For the model of thermal burn, we recorded a perivascular inflammation in the deep dermis and also at the border with the hypodermis.

On day 21 of the study, for the NC group, we observed an inflammatory infiltrate present in the hypodermis for all types of lesions and, in addition, tissue fibrosis for thermal burn. In thermal burn, dermal acanthosis was noted, as previously seen. For the NC group, we recorded the following aspects: in the incision model, the presence of edema and lymphocytes in the hypodermis; in excision, important congestion in the hypodermis and inflammation in the muscular layer, as well as numerous lymphocytes; and for thermal burn, lymphocytes in the superficial and perivascular dermis. For the OB group, we noted an inflammatory infiltrate in the hypodermis, in moderate amount, and perivascular lymphocytes in the incision model; in the excision-type lesion, we noted congestion in the hypodermis; for thermal burn, we noted the presence of lymphocytes in the hypodermis and vessels with thickened wall with perivascular lymphocytes.

For the group treated with honey-based ointment on the excision model, more than two-thirds of the dermis was represented by mature granulation tissue. In the incision model, lesions were observed, namely the discrete horizontalization of some collagen fibers in the subepidermal region. In thermal burns, less than two-thirds of the epidermis was represented by mature granular tissue (approximately fibrosis). The epidermis had an almost normal appearance, rectilinear, with areas of atrophy in all three models. 

For the group treated with propolis ointment, on day 21 of the experiment, in the excision model more than two-thirds of the dermis was a mature granular tissue with fibrosis. In the incision model, a quasinormal appearance of the epidermis was noted with 4–5 lymphocytes/HPF and 4–5 fibroblasts/HPF in the superficial dermis. In the thermal burn, the superficial third and the deep third of the dermis were represented by mature granulation tissue with slightly increased collagenization, sometimes with aspects of fibrosis, while the middle third of the dermis was characterized by collagenization (collagen densification).

In the batch treated with apilarnil-based ointment, on day 21, normal skin appendages were noted for dermal incision, collagenization and associated lymphocytic infiltrate were highlighted for excision model and dermal collagenization was noted for the thermal injury. 

For the group treated with ointment based on total bee products, on day 21 of the experiment, dermal collagenization and rare lymphocytes around hair follicles of normal appearance were observed for the incision model. At the same time, rare lymphocytes were noted in the excision model and collagenization was noted in the heat burn model.

## 3. Discussion

Regarding the rheological characterization of ointments, the amplitude sweep shows the structural stability of the samples at low values of deformation at both temperatures. At the temperature of 25 °C, the samples have a high rigidity and the structure is more sensitive to deformations. As the temperature rises, the structure becomes more flexible and the ointments have a soft texture and a better spreadability on the skin [8]. 

In the frequency sweep, the storage modulus G’ is higher than loss modulus G” over the entire frequency range, so the analyzed samples show a solid-like behavior at both temperatures (Figure 3). Dynamic moduli are almost parallel and have a small frequency dependence suggesting the presence of a stable three-dimensional network due to the physical interactions. At physiological temperature, the dynamic moduli decrease, which suggests that samples are softer and have a good spreadability on the skin [9].

The structural stability and the degree of fluidization of the ointments at physiological temperature were highlighted by the temperature test at constant amplitude and constant frequency (Figure 4). Time tests performed at both temperatures (25 and 37 °C) at constant amplitude and frequency showed the structural stability over time of the analyzed ointments (Figure 5). Moreover, the structural stability of the samples was highlighted by the flow curves (Figure 6) in which the high values of viscosity at low shear rate are proof of structural stability [8,10]. 

The chosen combination of ingredients in the final formulation has to provide the user with the most efficient use of the entrapped active principle and the best possible sensation. This means a well-defined stable structure with good flowing characteristics when applied and ability to recover the structure after application. Ointments’ rheological properties offer valuable information regarding their spreadability on the skin and the ability to release the active principle. The ointments are designed to exhibit long-term structure stability and the ability to ensure easy and uniform distribution and release of the active principle on the skin as well as good penetration in the wound. All these properties are evaluated through the rheological measurements that proved to be a useful tool for the appreciation of both mechanical and sensory properties of the product [8].

The HPLC analysis revealed the presence of various polyphenolic compounds in the propolis extract. Different studies demonstrated that topical administration of ferulic acid preparation promoted wound healing in diabetic rats by stimulating epithelialization and enhancing the levels of collagen markers, hydroxyproline and hexosamine, in the diabetic wound [11,12]. Ferulic acid also helped the healing process by inhibiting lipid peroxidation and increasing the levels of antioxidant enzymes, as well as serum zinc and copper levels [12]. 

The healing effect of p-coumaric acid was verified in a chronic model of ulcer in rats and also in the scratch assay where it stimulated the proliferation of fibroblasts cells [13]. Moreover, a p-coumaric acid biofilm design for wound dressing proved antibacterial action against common pathogens, free radical scavenging activity and anti-inflammatory effect by reducing matrix metallopeptidase-9 levels in a mouse wound model [14].

Song et al. [15] demonstrated the beneficial effect of caffeic acid on dermal wound healing in mice: treatment with caffeic acid increased collagen-like polymer levels in incised-wound tissue, as well as in NIH 3T3 fibroblast cells, and exhibited significant anti-inflammatory effect by regulating myeloperoxidase activity and decreasing lipid peroxidation and phospholipase A2 activity in the skin-incised tissue [15]. Although our analysis did not include caffeic acid phenethyl ester as a standard, this is one of the most important bioactive components of propolis, with anti-inflammatory, radical scavenging, antimicrobial, immunomodulatory and wound healing properties [16].

The wound healing activity of flavonoids is well known, many such compounds being already tested on animal models of acute or chronic wounds with good results: quercetin, kaempferol, luteolin, apigenin, rutoside and chrysin—another important flavonoid found in propolis and honey [17]. Flavonoids promote epithelialization, accelerate wound contraction rates, stimulate angiogenesis, reduce oxidative stress and modulate the inflammatory response. Experiments showed that flavonoids increase proliferation, differentiation and secretion of keratinocytes; augment expression of matrix metalloproteinase-9 which is essential for wound re-epithelialization; and promote migration of fibroblasts [17].

The identified polyphenols, especially those found in high amounts such as ferulic, p-coumaric and caffeic acids, contributed to the wound healing effect of propolis-based ointment.

The inclusion of honey in the formulation of the ointment was based on several considerations: on the skin, glucose, present in honey in large quantities, diffuses freely in the interstitial fluid of the dermis and epidermis, from where it reaches the cells [18]. More than half of the glucose absorbed by the skin is used by the epidermis, the intake being higher in the basal layer than in other layers [19]. Moreover, numerous carbohydrates, including a wide variety of hexoses, as well as sialic acid, are part of many subcellular structures of keratinocytes in all layers. Thus, carbohydrates and their derivatives enter into the composition of cellular organelles, plasma membrane, basement membrane and glycocalyx [18]. Besides, there are studies in which the topical use of honey caused the remission of chemically induced erythema [20]. The use of honey in the case of a chemical burn has led to very good effects in terms of healing and re-epithelialization of severely affected tissues [21]. 

Glycoproteins are compounds found in many skin structures: plasma membrane, desmosomes, basement membrane, lamellar bodies, the Golgi apparatus of keratinocytes and even in melanosomes. A series of glycoproteins are attached to the surface of epidermal cells, and they contain carbohydrates with six carbon atoms: mannose, galactose, fucose and N-acetyl glucosamine. Mannose, galactose and N-acetylglucosamine are found on all living epidermal cells, while fucose is found only on the granular layer, and N-acetyl galactosamine is found on spinous and granular cells [18]. Literature data show the benefit of using honey in wound healing: it has a very good diffusion rate in the skin and also stimulates the growth of the affected tissue, accelerates healing and produces debridement [22]. In debridement, honey acts synergistically with another apitherapy product, propolis, as observed for the present study on day 6 for incision, day 9 for excision and day 12 for burn. 

It should also be added that honey is an effective antimicrobial due to its acidity (given by organic acids, especially gluconic acid); its high sugar content, which exerts osmotic pressure on bacterial cells; the presence of antibacterial peptides (defensin-1), enzymes and phytochemicals such as polyphenols; and the enzymatic release of hydrogen peroxide in honey, with antibiotic action [23]. These compounds intervened beneficially in the inflammatory process and edema, which reached their peak on the third day after induction of the lesion, especially in the case of the excision and burn models. In addition, the hygroscopic capacity of honey has contributed to a significant reduction in edema. In clinical practice, topical honey treatment promoted cell and tissue regeneration and had antiseptic, anti-inflammatory and analgesic actions (alleviating pain in the affected region) [22,23]. For the batch treated with honey ointment, dermal collagenization was observed, as for the batch treated with propolis, but not of the same intensity, which intervenes favorably in the healing process.

Moreover, the presence of vitamin C in honey stimulates growth and repair of damaged tissues. Vitamin C participates in collagen synthesis which is crucial for the healing/regeneration process of lesions [24]. In the dermis, vitamin C acts mainly on fibroblasts by increasing the levels of mRNA for procollagen and by enhancing the transcription of genes responsible for the synthesis of collagen of types I and III [25]. Vitamin C is a cofactor for two crucial enzymes in collagen synthesis: proline and lysine hydroxylases that stabilize the tertiary structure of the collagen molecule and give it structural strength [24]. As a powerful antioxidant, vitamin C protects the skin against UV radiation and photoaging [26]. Patients with inflammatory skin diseases, such as psoriasis and atopic dermatitis, which involve the production of reactive oxygen species, have low concentrations of vitamin C. Decreased vitamin C levels in the dermis are also determined by increasing age, and topical application of vitamin C causes changes in skin appearance and ultrastructure, suggesting a positive role in preventing and treating skin aging [26]. It is possible that the very presence of vitamin C in honey ointment is responsible for the nonvicious healing of the skin tissue, which does not present keloid scars in any of the lesion models targeted by this study. Vitamin C also increases keratinocyte differentiation and formation of the stratum corneum and intervenes in the function of the epidermal barrier by increasing the synthesis of ceramides [24]. 

The effectiveness of propolis ointment for the treatment of skin tissue lesions was confirmed in a study by Pressolato et al. [27], who demonstrated the beneficial action of propolis on promoting lesion debridement. They deduced that the propolis ointment stimulated the production of collagen fibers, proving its efficiency in the treatment of burns. An imbalance of collagenization or the absence of collagen will negatively affect the healing [28]. The efficacy of propolis incorporated in the ointment formula can be attributed to the presence of caffeic acid phenethyl ester (CAPE), which accelerates wound healing by reducing inflammatory parameters and oxidative damage at the site of burn injury [29]. In addition to the antioxidant and anti-inflammatory activities, CAPE stimulates collagen deposition, re-epithelialization and wound closure of pressure ulcers in mice [30]. The topical use of propolis in the case of chemically induced skin erythema has led to favorable results in terms of healing and re-epithelialization [20]. 

It is important to note that the use of propolis as such leads to normal clinical healing and re-epithelialization, but microscopically it was revealed that it produces a marked collagenization that is favorable in the first days of the healing process but can ultimately compromise the normal repair process. Normal collagen production during the healing process is essential for epithelial migration and postinjury proliferation, but at the end of the healing process, too high a level of collagen can compromise the end result of the injury [28]. We incorporated propolis into two of the formulations resulting in the presence of normal collagenization, as well as good clinical effects. Moreover, some clinical studies reported the efficacy of propolis in treating burns and diabetic ulcers. For example, topical application of propolis ointment led to faster healing of superficial second-degree burns than the use of conventional treatment with silver sulfadiazine in the ambulatory care setting [31]. In a pilot study, topical propolis applied weekly accelerated wound closure and was well tolerated in diabetic foot ulcer treatment [32]. Similarly, ulcerations of patients with nonhealing venous leg ulcers healed completely after 6 weeks of topical therapy with 7% propolis ointment [33].

The nutritional and apitherapeutic value of drone brood homogenate is given by the food received and existing in the cells: honey, bee bread and glandular secretions of nurse bees [34].

The total apitherapy preparation ensures the synergistic presence of apitherapy principles with a healing and regenerating effect on cells and tissues; it includes active principles with bactericidal, antifungal, anti-inflammatory and antioxidant properties. In addition, it contains compounds with toning, biostimulating action which ensures the elasticity and flexibility of the skin tissue and compounds with the stimulating effect on the enzyme system, while also acting as a growth factor. 

The ointment based on a mixture of bee products has many advantages: honey, propolis, and apilarnil are biocompatible natural products; it uses a preparation temperature not exceeding 40 °C; all the biological characteristics of the apitherapy compounds used are preserved and no toxic residues remain due to the technological processing; the base for incorporating the bee products has 50% lanolin (a product of natural origin) and 50% pharmaceutical vaseline, being preferred to the simple base (10% lanolin and 90% pharmaceutical vaseline) because it provides a therapeutic response; and no preservatives or other synthetic compounds are used. Bee products are biocompatible with the human body and have no side effects such as synthetic compounds. Last but not least, the apitherapy ointment ensures great simplicity and ease of use; the administration can be done on an outpatient basis, following the therapeutic recommendation. Due to the complexity of the active principles present in the tested preparation, the healing time when using the apitherapy ointment is shorter, with favorable results in terms of healing.

## 4. Materials and Methods

### 4.1. Chromatographi Conditions for HPLC Analysis 

#### 4.1.1. Polyphenol Analysis

Polyphenolic compounds in hydroalcoholic propolis extract, oily propolis extract and honey sample were quantified using a previously described HPLC-UV-MS method [35]. Eighteen external standards were used: caffeic acid, chlorogenic acid, p-coumaric acid, kaempferol, apigenin, rutin, quercetin, quercitrin, isoquercitrin, fisetin, hyperoside, myricetin (Sigma, Neustadt, Germany), ferulic acid, gentisic acid, sinapic acid, patuletin, luteolin (Roth, Germany) and caftaric acid (Dalton, Toronto, ON, Canada). Calibration curves in the 0.5–50 μg/mL range with good linearity (R^2^ > 0.999) were used to determine the concentration of polyphenols in the sample. 

#### 4.1.2. Caffeic and Chlorogenic Acids Analysis

Caffeic and chlorogenic acid could not be quantified in the hydroalcoholic propolis extract due to overlapping, but they were identified in MS detection (qualitative analysis) based on differences between their molecular mass and MS spectra. In order to separate and quantify these two hydroxycinnamic acids, a new analysis was conducted under different chromatographic conditions, as described before [7]. Calibration curves of caffeic and chlorogenic acids in the range of selected concentrations (0.06–4 μg/mL) showed a good linear correlation coefficient (R^2^ > 0.99).

#### 4.1.3. Methoxylated Flavonoids Analysis

Methoxylated flavonoids were quantified in the hydroalcoholic propolis extract using a previously reported LC-MS method [35]. Six standards were used: jaceosidin, eupatilin (ALB Technology, Hong Kong, China), casticin, acacetin, eupatorin and hispidulin (Sigma, Neustadt, Germany). Calibration curves in the 0.02–6 μg/mL range with good linearity (R^2^ > 0.99) were used to determine the concentration of methoxylated flavones.

### 4.2. Ointments Preparation

#### 4.2.1. The Ointment with Propolis Extract

In the ointment base (50 g vaseline and 50 g lanolin), 15 mL of propolis oily extract and 15 mL of propolis hydroalcoholic extract were gradually incorporated on water bath at 40 °C. 

The hydroalcoholic propolis extract was obtained by macerating the propolis in 70% *v*/*v* ethanol (ratio propolis: solvent of extraction 1:10) for 72 h at room temperature, away from light. Subsequently, it was centrifuged at 3000 rpm and then filtered. The propolis oily extract was obtained by macerating the crushed propolis in virgin olive oil, on a magnetic stirrer, at a maximum temperature of 40 °C, for 7 days, away from light.

#### 4.2.2. The Ointment with Honey

In the ointment base, 50 g of honey was incorporated, on water bath, at a temperature of 40 °C.

#### 4.2.3. The Ointment with Apilarnil

In the ointment base, 2 g of lyophilized apilarnil was gradually incorporated, on water bath, at a temperature of 40 °C.

#### 4.2.4. The Ointment with a Mixture of Bee Products

The total apitherapy ointment was prepared by mixing the three previously described ointments in equal parts.

The chosen concentrations of propolis, honey and apilarnil in the ointments were selected based on previous studies carried out on wound healing [20,21,36]. We observed both clinically and microscopically that these are the optimal amounts for positive effects on the treatment of incision, excision and heat burn lesions.

### 4.3. Chemical Reagents and Bee Products

The ethyl alcohol was purchased from Sigma-Aldrich (Steinheim, Germany), while lanolin and vaseline were from Farma Chim (Ploiesti, Romania). 

The samples of honey, apilarnil and propolis were collected from the same apiary, S.C. Stupina SRL, which is located in Balanesti, Gorj County, Romania. The samples were taken in 2016, as follows: honey in August, apilarnil in April, propolis in April–September, every 25th of the month, making a mixture of the samples taken.

The organoleptic analysis of honey (appearance, consistency, color, taste and odor) and physicochemical determinations of honey (free acidity, reducing sugar, easily hydrolyzable sugar, hydroxymethylfurfural, diastase index, ash, water) and apilarnil (total protein, total fats, ash and water content) were performed at ICDA Bucharest (Research and Development Institute for Beekeeping), and the results are included in the Supplementary Information file (Tables S2 and S3). Vitamin C concentration was determined by an adapted titrimetric method [37], and the result is recorded in the Supplementary file.

### 4.4. Rheological Characterization of Ointments

Experimental determination was performed on the interdisciplinary training and research platform “High performance multifunctional polymeric materials for medicine, pharmacy, microelectronics, energy/information storage, environment protection” (MATMIP) acting within the Natural and Synthetic Polymers Department of “Cristofor Simionescu” Faculty of Chemical Engineering and Environmental Protection of “Gheorghe Asachi” Technical University of Iasi. All experiments were conducted on the modular rheometer Anton Paar Physica MCR 501 equipped with a Peltier system for temperature adjustment and a serrated parallel plate 50 mm in diameter. Oscillatory and rotational tests were performed (amplitude sweep, frequency sweep, temperature test, time test and flow curve) at 25 and 37 °C. 

### 4.5. Experimental Skin Lesions 

The experiment was performed on Wistar adult male rats with a body weight of 220–250 g. The animals were housed in a bright, temperature-controlled room with dark–light cycles of 12:12 h, where the temperature (22 ± 0.5 °C) and relative humidity (65–70%) were kept constant.

The study included six groups with 7 Wistar rats per group as follows: NC group (negative control group—not treated), OB group—ointment base group (treated with the ointment base), Ho group (treated with honey ointment), Ap group (treated with apilarnil ointment), Pr group (treated with propolis ointment), To group (treated with the ointment based on the mixture of bee products).

Before performing the experimental models, the animals were anesthetized with ketamine ip (100 mg/kg), and the hair on the back was shaved and cleaned with 70% alcohol.

Three models of skin lesions (linear incision, circular excision, thermal burn) were performed according to the models documented in the literature [7,38,39]. Ointments and ointment base were applied topically (0.5 g), once a day, for 21 days.

#### 4.5.1. Evaluated Parameters

Clinical and macroscopic evaluation was performed throughout the study, recording the re-epithelialization period; determining the rate of contraction of the lesion (days 6 and 9); and performing the histopathological examination on days 1, 3, 12 and 21 of the treatment.

##### Wound Contraction Rate Measurement (WCR)

The calculation of the circular lesion area (wound excision) was determined with the formula A = πr^2^.

The calculation of the elliptical area resulting from the process of contraction of the lesion was calculated according to the formula πa × b/4, where a is the major axis and b is the minor axis.

The wound contraction rate (WCR) was calculated as a percentage of the original wound size (A_0_ = 50.27 mm^2^) for each animal according to the formula WCR = (A_0_ − A_t_)/A_0_ × 100, where A_t_ = the wound size on days 6 and 9.

##### Measurement of the Re-Epithelialization Period

The closure of the lesion was considered as the endpoint of complete epithelialization, and the days required for this process were considered as the epithelialization period.

##### Histopathological Examination

In order to take a skin fragment to detect the epithelialization process, the animals were anesthetized intraperitoneally with ketamine (100 mg/kg). The samples collected were processed according to the method described above [6].

The analysis of inflammatory elements based on the papillary and reticular dermis, hypodermis and striated muscle tissue was based mainly on a scoring system in the literature [40,41], adapted to the research requirements. This scoring system numerically quantifies lymphocytes/plasma cells. Following all the elements associated with the inflammatory process—vasodilation, congestion, edema, collagen changes and fibrosis—allowed a detailed description of the morphological picture in all groups analyzed. 

For this purpose, five microscopic fields with a magnification of 400× were observed at the level of the papillary and reticular dermis and also at the hypodermis and striated muscle tissue and chosen as representative for each case. The value of the final score was represented by the average value of the five fields observed. A score of 0 (S0) represented no inflammatory infiltrate, a score of 1 (S1) represented mild/rare/occasional inflammatory infiltrate (<10 lymphocytes/HPF), a score of 2 (S2) represented moderate/focal inflammatory infiltrate (11–30 lymphocytes/HPF) and a score of 3 (S3) represented severe inflammatory infiltrate (numerous lymphocytes > 30/HPF).

The evaluation of the thermal burn depths (D) was also achieved based on adapted scores from the literature as follows: D0—normal skin; D1—epithelial necrosis within the epidermis, the basement membrane remains intact; D2—necrosis of the epidermis and basement membrane, skin appendage remains intact; D3—necrosis of skin appendages and dermal connective tissue; D4—extensive necrosis within the hypodermic tissue [42,43].

Gradation of infection (G) was based on an adapted score from the literature [44,45], as follows: G0—no microorganisms are identified in the sections, GI—microorganisms are identified on the surface of the lesion, GIa—several microbial elements are highlighted, GIb—many microbial elements are highlighted, GII—microorganisms affect the superficial dermis, GIII—microorganisms affect the entire thickness of the dermis, GIV—microorganisms extensively affect the adjacent viable tissues and the hypodermis.

### 4.6. Statistical Analysis

The data obtained from the wound excision model were analyzed by one-way ANOVA followed by the Bonferroni post-test. The statistical analysis was performed using SPSS 15, where *p* < 0.05 was considered statistically significant.

## 5. Conclusions

Rheological tests conducted at 25 and 37 °C for the ointments provided valuable information about their structural properties and temperature and time stability. At 37 °C, the structure is flexible for all analyzed ointments (the values of dynamic moduli decrease), suggesting a good spreadability on the skin and allowing better absorption. Knowledge of the rheological behavior of ointments can be used to predict the efficiency of different products and the possibility to use them for specific applications.

Each of the apitherapy formulations based on propolis, honey and apilarnil, separately, has a healing and re-epithelialization effect on the three experimental models. To our knowledge, this is the first study that evaluated the healing property of drone brood homogenate in excision, incision and thermal wound models. The ointment that brought together the three bee products was proven to have synergistic activity and resulted in a shorter healing period of the lesions, this aspect being obvious clinically; the lesions evolved towards healing that did not involve the result of a keloid scar, as it happened for the untreated batch. Moreover, good results were obtained from a histopathological point of view, namely vascularization via the process of angiogenesis being resumed.

Through the cumulative presence of the active principles of the three bee products with healing properties and cellular and tissue regenerating effect, the preparation used had tonic, regenerating, biostimulating action and ensured the elasticity and flexibility of skin tissue. In addition to healing, the result was also esthetically satisfying, an important objective especially in the case of burn injuries.

## Figures and Tables

**Table 1:** Concentration of phenolic compounds (µg/mL) in the hydroalcoholic propolis extract.

Compound	Concentration in Extract (µg/mL)
p-Coumaric acid	1516.119 ± 60.64 (*n* = 3)
Ferulic acid	1771.669 ± 53.15 (*n* = 3)
Isoquercitrin	1.009 ± 0.08 (*n* = 3)
Rutoside	0.823 ± 0.05 (*n* = 3)
Quercitrin	11.573 ± 0.57 (*n* = 3)
Quercetin	18.295 ± 0.73 (*n* = 3)
Luteolin	6.604 ± 0.46 (*n* = 3)
Kaempferol	19.537 ± 0.97 (*n* = 3)
Apigenin	19.195 ± 0.57 (*n* = 3)
Caffeic acid	796.40 ± 31.85 (*n* = 3)
Chlorogenic acid	1.670 ± 0.11 (*n* = 3)
Acacetin	0.613 ± 0.03 (*n* = 3)
Jaceosidin	0.821 ± 0.04 (*n* = 3)
Hispidulin	0.393 ± 0.03 (*n* = 3)

**Table 2:** Evaluation of wound area, re-epithelialization area and wound contraction rate (WCR) for the excision model.

Experimental Groups	Wound Area (mm^2^)			Re-Epithelialization Area (mm^2^)	WCR (%) (Mean ± SEM)	
	Day 0	Day 6	Day 9	Day 12	Day 6	Day 9
NC Group	64.0 (8.0 × 8.0)	61.6 (7.7 × 8.0)	56.25 (7.5 × 7.5)	-	5.78 ± 1.86	14.85 ± 2.95
OB Group	64.0 (8.0 × 8.0)	45.5 (6.5 × 7.0)	40.8 (6.0 × 6.8)	-	26.74 ± 2.13 *	33.79 ± 2.21 *
Ho Group	64.0 (8.0 × 8.0)	20.0 (4.0 × 5.0)	4.0 (2.0 × 2.0)	1.5 × 2.0	69.38 ± 0.63	93.65 ± 0.28
Pr Group	64.0 (8.0 × 8.0)	16.0 (4.0 × 4.0)	1.5 (1.0 × 1.5)	1.0 × 1.0	73.06 ± 1.36	97.97 ± 0.16
Ap Group	64.0 (8.0 × 8.0)	25.0 (5.0 × 5.0)	9.0 (3.0 × 3.0)	2.0 × 2.0	63.93 ± 2.26	84.69 ± 0.68
Ta Group	64.0 (8.0 × 8.0)	18.0 (4.0 × 4.5)	2.0 (1.0 × 2.0)	1.0 × 1.2	72.50 ± 0.36	96.54 ± 0.18

* *p* < 0.001. NC group (negative control group—not treated), OB group—ointment base group (treated with the ointment base), Ho group (treated with honey ointment), Pr group (treated with propolis ointment), Ap group (treated with apilarnil ointment), Ta group (treated with the ointment based on mixture of bee products).

**Table 3:** Histopathological evaluation of the tissue samples on day 1.

Incision		
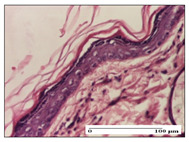	** 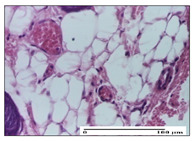 **	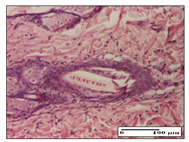
lymphocytes, epidermis and dermis (S1, G0)	vascular congestion, hypodermis (S0, G0)	rare periadnexal inflammatory infiltrate (S1, G0)
Excision		
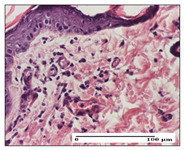	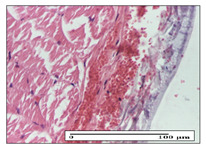	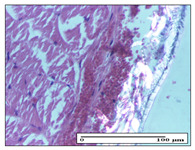
slight edema, perivascular lymphocytes (S2, G0)	hemorrhage in the muscular layer (S0, G0)	hemorrhage in the muscular layer in polarized light microscopy (S0, G0)
Thermal Burn		
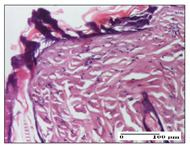	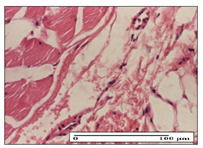	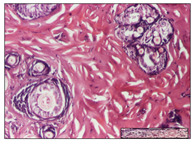
severe dermal collagenization (S1, D1, G0)	congestion in hypodermis (S0, D0, G0)	severe dermal collagenization (S0, D0, G0)

Inflammatory infiltration scoring: S0 (no inflammatory infiltrate), S1 (mild inflammatory infiltrate), S2 (moderate inflammatory infiltrate), S3 (severe inflammatory infiltrate). The thermal burn depths (D): D0—normal skin, D1—epithelial necrosis within the epidermis, the basement membrane remains intact; D2—necrosis of the epidermis and basement membrane, skin appendages remain intact; D3—necrosis of skin appendages and dermal connective tissue; D4—extensive necrosis within the hypodermic tissue. Gradation of infection (G): G0—no microorganisms are identified in the sections, GI—microorganisms are identified on the surface of the lesion, GI_a_—several microbial elements are highlighted, GI_b_—many microbial elements are highlighted, GII—microorganisms affect the superficial dermis, GIII—microorganisms affect the entire thickness of the dermis, GIV—microorganisms extensively affect the adjacent viable tissues and the hypodermis.

**Table 4:** Histopathological evaluation of the tissue samples on day 3.

Experimental Groups	Incision	Excision	Thermal Burn
NC Group	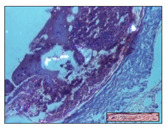	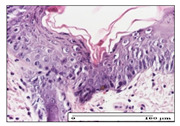	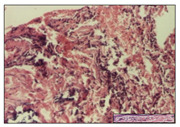
	abscess in keratin layer—polarized light microscopy (S3, GI_a_)	acanthosis and vacuolar degeneration, epidermis and dermis (S1, G0)	severe inflammation with hemorrhagic areas (S3, D2, G0)
OB Group	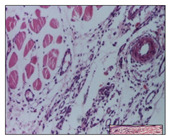	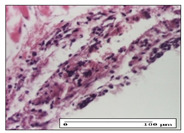	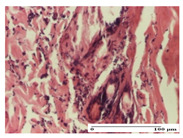
	inflammatory infiltrate in hypodermis (S2, G0)	important inflammatory infiltrate in hypodermis (S2, G0)	severe inflammation with hemorrhagic areas (S2, G0)
Ho Group	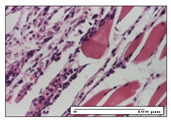	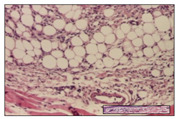	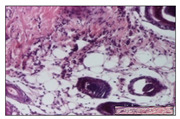
	inflammatory infiltrate in hypodermis and interstitial muscle (S2, G0)	inflammatory infiltrate in hypodermis (S2, G0)	inflammation in hypodermis (S2, D0, G0)
Pr Group	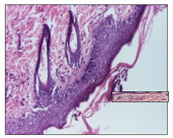	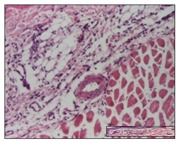	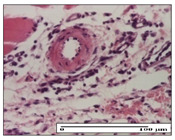
	edema in superficial dermis (S1, G0)	inflammatory infiltrate in hypodermis (S2, D0, G0)	perivascular inflammatory infiltrate (S2, D0, G0)
Ap Group	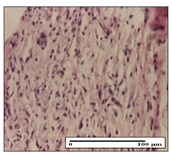	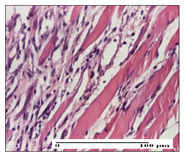	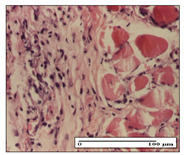
	fibroblasts (S1, G0)	interstitial inflammatory infiltrate, striated muscle (S1, G0)	myositis, moderate inflammatory infiltrate in the muscle interstitium (S1, D0, G0)
Ta Group	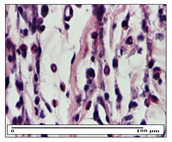	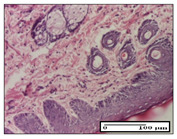	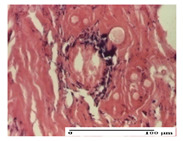
	inflammation in hypodermis (S1, G0)	lymphocytes in superficial dermis (S0, G0)	severe inflammation with hemorrhagic areas (S3, D0, G0)

Inflammatory infiltration scoring: S0 (no inflammatory infiltrate), S1 (mild inflammatory infiltrate), S2 (moderate inflammatory infiltrate), S3 (severe inflammatory infiltrate). The thermal burn depths (D): D0—normal skin, D1—epithelial necrosis within the epidermis, the basement membrane remains intact; D2—necrosis of the epidermis and basement membrane, skin appendage remains intact; D3—necrosis of skin appendages and dermal connective tissue; D4—extensive necrosis within the hypodermic tissue. Gradation of infection (G): G0—no microorganisms are identified in the sections, GI—microorganisms are identified on the surface of the lesion, GI_a_—several microbial elements are highlighted, GI_b_—many microbial elements are highlighted, GII—microorganisms affect the superficial dermis, GIII—microorganisms affect the entire thickness of the dermis, GIV—microorganisms extensively affect the adjacent viable tissues and the hypodermis.

**Table 5:** Histopathological evaluation of the tissue samples on day 12.

Experimental Groups	Incision	Excision	Thermal Burn
NC Group	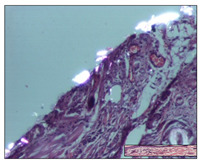	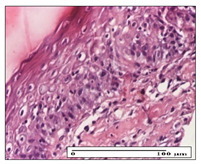	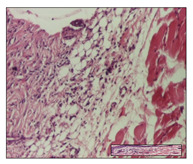
	congestion in hypodermis—detail in polarized light microscopy (S1, G0)	exocytosis (S2, G0)	inflammatory infiltrate in hypodermis (S2, D0, G0)
OB Group	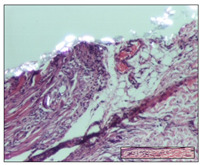	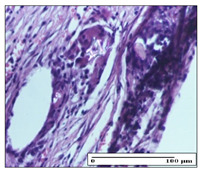	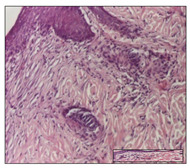
	multinucleated foreign-body giant cell, congestion in hypodermis—detail in polarized light microscopy (S2, G0)	multinucleated foreign-body giant cell—detail in polarized light microscopy (S2, G0)	rare periadnexal inflammatory infiltrate (S2, D0, G0)
Ho Group	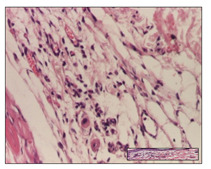	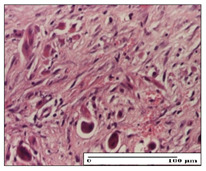	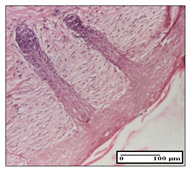
	rare lymphocytes in hypodermis (S1, G0)	mature granulation tissue, residual myocytes in the area of connective tissue organization (S1, G0)	dermal collagenization(S0, D0, G0)
Pr Group	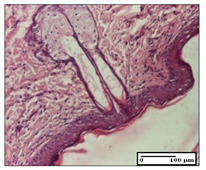	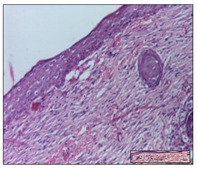	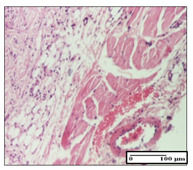
	collagenization in superficial dermis (S1, G0)	dermal collagenization with congestion (S1, G0)	moderate hypodermic infiltrate (S1, D0, G0)
Ap Group	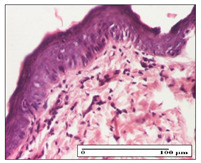	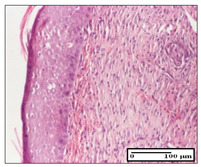	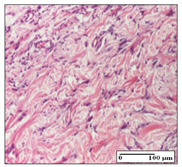
	rare perivascular lymphocytes (S1, G0)	severe dermal collagenization (S1, G0)	dermal collagenization (S1, D0, G0)
Ta Group	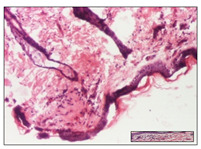	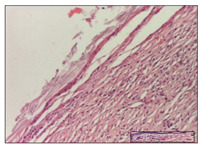	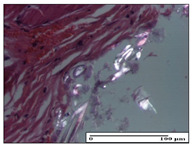
	rare lymphocytes in superficial dermis (S1, G0)	dermal collagenization (S1, G0)	inflammatory infiltrate—detail in polarized light microscopy (S1, D0, G0)

Inflammatory infiltration scoring: S0 (no inflammatory infiltrate), S1 (mild inflammatory infiltrate), S2 (moderate inflammatory infiltrate), S3 (severe inflammatory infiltrate). The thermal burn depths (D): D0—normal skin, D1—epithelial necrosis within the epidermis, the basement membrane remains intact; D2—necrosis of the epidermis and basement membrane, skin appendage remains intact; D3—necrosis of skin appendages and dermal connective tissue; D4—extensive necrosis within the hypodermic tissue. Gradation of infection (G): G0—no microorganisms are identified in the sections, GI—microorganisms are identified on the surface of the lesion, GI_a_—several microbial elements are highlighted, GI_b_—many microbial elements are highlighted, GII—microorganisms affect the superficial dermis, GIII—microorganisms affect the entire thickness of the dermis, GIV—microorganisms extensively affect the adjacent viable tissues and the hypodermis.

**Table 6:** Histopathological evaluation of the tissue samples on day 21.

Experimental Groups	Incision	Excision	Thermal Burn
NC Group	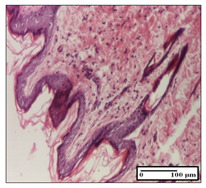	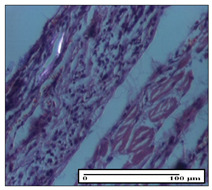	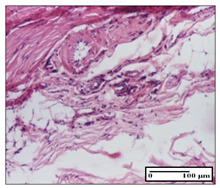
	edema and inflammatory infiltrate (S1, G0)	inflammation in muscular layer—detail in polarized light microscopy (S1, G0)	lymphocytes in hypodermis (S1, D0, G0)
OB Group	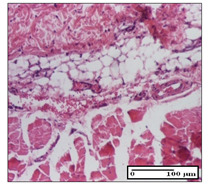	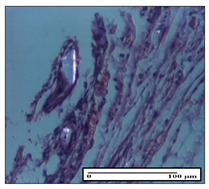	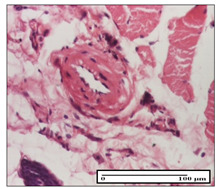
	hypodermis with inflammatory infiltrate (S0, D0)	hypodermis with inflammatory infiltrate—detail in polarized light microscopy (S1, G0)	vessel with thickened wall, lymphocytes (S0, D0, G0)
Ho Group	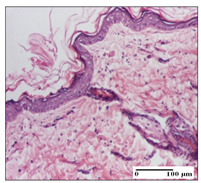	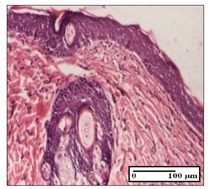	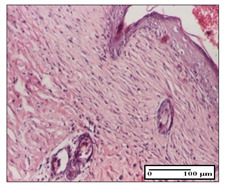
	slight dermal edema (S0, G0)	dermal collagenization (S0, G0)	severe dermal collagenization (S1, D0, G0)
Pr Group	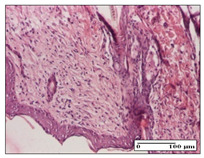	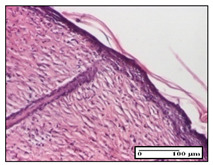	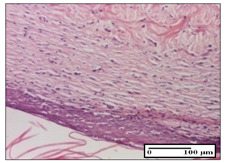
	dermal collagenization (S1, G0)	severe dermal collagenization (S1, G0)	severe dermal collagenization (S0, D0, G0)
Ap Group	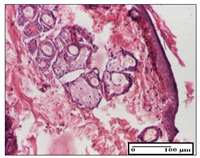	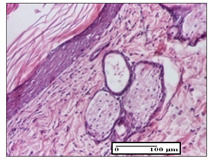	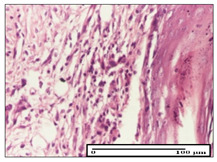
	skin appendages (S0, G0)	dermal collagenization and associated inflammatory infiltrate (S0, G0)	moderate inflammatory infiltrate in superficial dermis (S1, D0, G0)
Ta Group	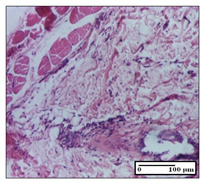	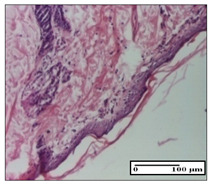	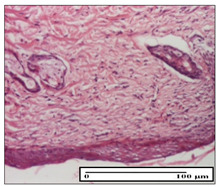
	hair follicle with rare lymphocytes (S1, G0)	rare lymphocytes (S1, G0)	severe dermal collagenization (S1, D0, G0)

Inflammatory infiltration scoring: S0 (no inflammatory infiltrate), S1 (mild inflammatory infiltrate), S2 (moderate inflammatory infiltrate), S3 (severe inflammatory infiltrate). The thermal burn depths (D): D0—normal skin, D1—epithelial necrosis within the epidermis, the basement membrane remains intact; D2—necrosis of the epidermis and basement membrane, skin appendage remains intact; D3—necrosis of skin appendages and dermal connective tissue; D4—extensive necrosis within the hypodermic tissue. Gradation of infection (G): G0—no microorganisms are identified in the sections, GI—microorganisms are identified on the surface of the lesion, GI_a_—several microbial elements are highlighted, GI_b_—many microbial elements are highlighted, GII—microorganisms affect the superficial dermis, GIII—microorganisms affect the entire thickness of the dermis, GIV—microorganisms extensively affect the adjacent viable tissues and the hypodermis.

## Data Availability

Data is contained within the article and supplementary files.

## Supplementary Materials

The following are available online at https://www.mdpi.com/article/10.3390/ph14111146/s1, Figure S1: UV and MS chromatograms of gentisic acid, myricetol and hyperoside, Figure S2: HPLC chromatogram of oily propolis extract (1-caffeic acid, 2-p-coumaric acid, 3-ferulic acid), Figure S3: UV and MS chromatograms of identified compounds in the oily propolis extract, Figure S4: HPLC chromatogram of honey sample (1-gentisic acid, qualitatively identified), Table S1: Polyphenolic compounds identified in the oily propolis extract, Table S2: Organoleptic analysis and physico-chemical determination of honey—Bulletin no. 945/12.12.2016 (ICDA Bucharest—Research and Development Institute for Beekeeping), Table S3: Physico-chemical determination of apilarnil—Bulletin no. 947/12.12.2016 (ICDA Bucharest—Research and Development Institute for Beekeeping).
