# Supplementary material for: Evaluation of the Healing Effect of Ointments Based on Bee Products on Cutaneous Lesions in Wistar Rats
Source: Pharmaceuticals (Basel). 2021 Nov 11;14(11):1146. doi: 10.3390/ph14111146 (PMC8618059; doi:10.3390/ph14111146)
Supplement: Supplementary file 1 [file pharmaceuticals-14-01146-s001.zip › pharmaceuticals-1426593-supplementary.pdf]

HPLC-UV-MS analysis of hydro-alcoholic propolis extract

The UV quantitation is made only if there is a match with MS spectra AND if the UV signal allows quantitation (S/N >5). For gentisic acid, hyperoside and myricetin those criteria were not met, so only qualitative results were provided. The UV, together with corresponding MS chromatograms for the 3 compounds (aligned to time axis) are shown below: (chromatograms presented: UV, MS-gentisic acid, MS-myricetol, MS-hyperoside).

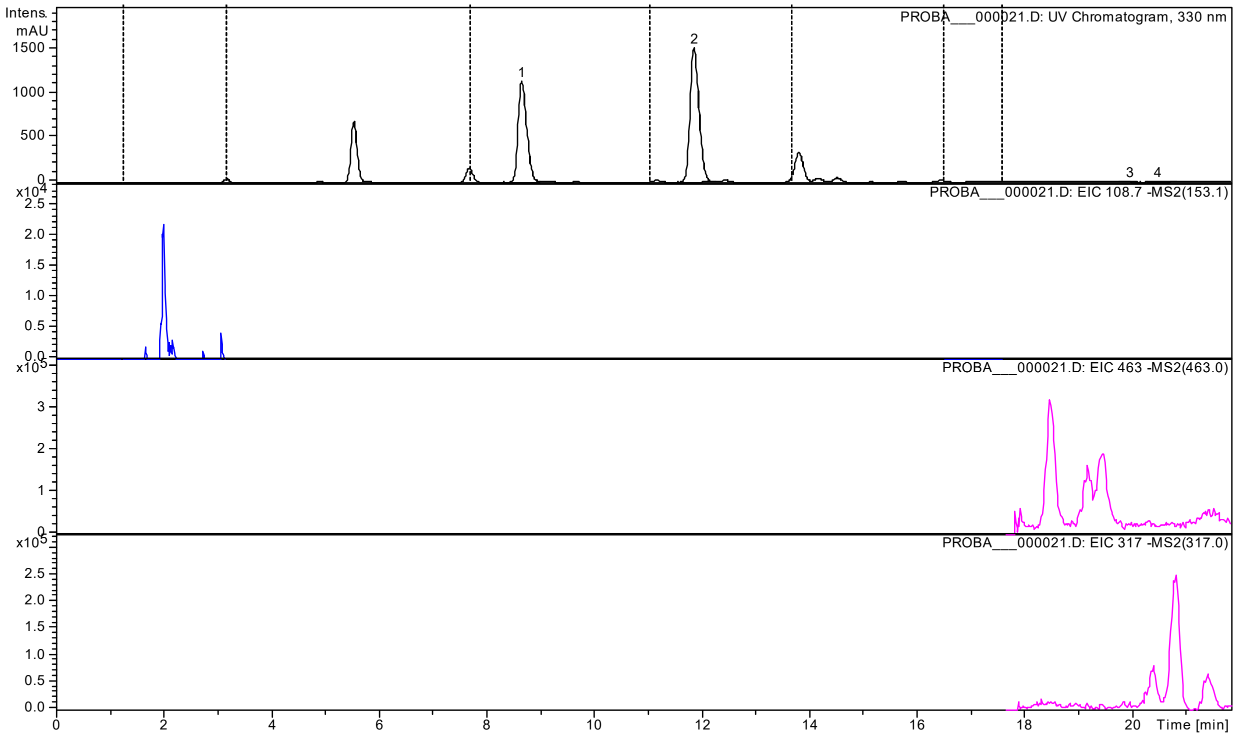

Figure S1. UV and MS chromatograms of gentisic acid, myricetol and hyperoside

HPLC-UV-MS analysis of oily propolis extract and honey sample

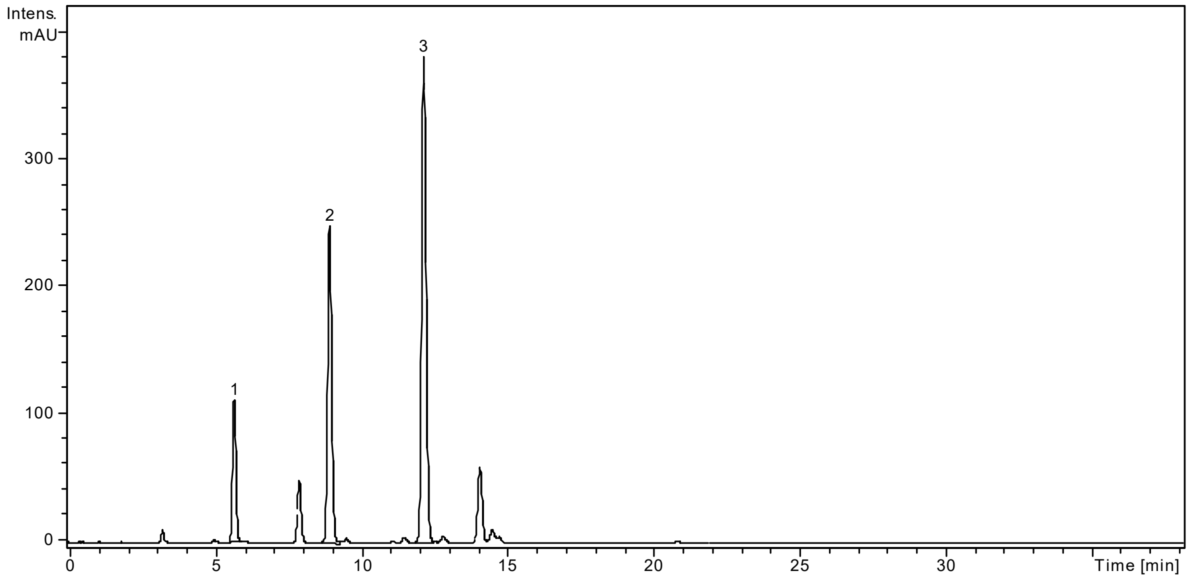

Figure S2. HPLC chromatogram of oily propolis extract (1-caffeic acid, 2-p-coumaric acid, 3-ferulic acid)

Table S1. Polyphenolic compounds identified in the oily propolis extract

No. on chromatogram	Compound	UV Identified	MS qualitatively identified	Concentration (μg/ml)
	Gentisic acid	NO	YES	qualitatively
1	Caffeic acid	YES	YES	19.129 ± 0.95
2	p-coumaric acid	YES	YES	71.661 ± 2.86
3	Ferulic acid	YES	YES	99.600 ± 2.98

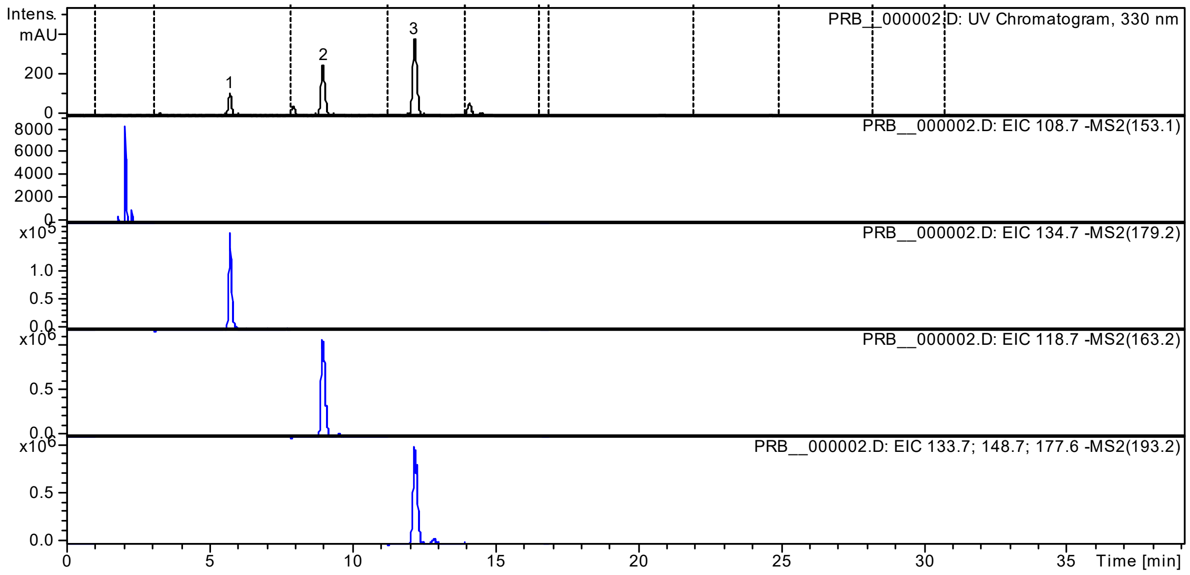

Figure S3. UV and MS chromatograms of identified compounds in the oily propolis extract

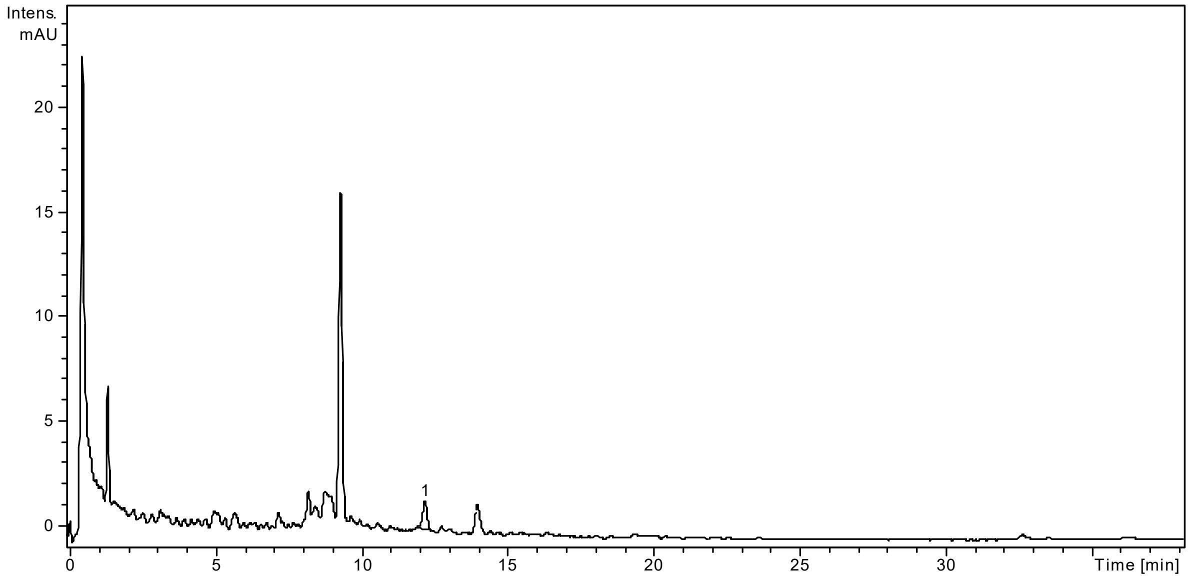

Figure S4. HPLC chromatogram of honey sample (1-gentisic acid, qualitatively identified)

Table S2. Organoleptic analysis and physico-chemical determination of honey – Bulletin no. 945/12.12.2016 (ICDA Bucharest - Research and Development Institute for Beekeeping)

Organoleptic properties of honey	Methods of analysis	Results
appearance	SR 784 / 3-2009	clean, homogeneous, without impurities, without foam
consistency	SR 784 / 3-2009	viscous mass, crystallized with coarse crystals
color	SR 784 / 3-2009	yellow
taste and odor	SR 784 / 3-2009	sweet taste, with a pleasant floral aroma

Physico-chemical determinations of honey	Methods of analysis	Results
free acidity	LCPA // DFC-PS-02 SR 784 / 3-2009 point 4.3	2.20 ± 0.351 mL NaOH 1N/100 g
reducing sugar expressed as invert sugar	LCPA // DFC-PS-06 // Elser SR 784 / 3-2009 pt.4.4	74.50 ± 0.7806 %, g/g

easily hydrolysable sugar expressed as sucrose	LCPA // DFC-PS-07 // Elser SR 784 / 3-2009 point 4.5	1.90 ± 0.1522 %, g/g
hydroxymethylfurfural	LCPA // DFC-PS-03 // Winkler SR 784 / 3-2009 point 4.9	1.44 ± 0.1713 mg/100 g
diastase index	LCPA // DFC-PS-05 // Gothe SR 784 / 3-2009 pt.4.7	29.40 ± 0.3596 Gothe units
ash	LCPA // DFC-PS-09	0.1022 ± 0.008 %, g/g
water	LCPA // DFC-PS-16 // refractometric method SR 784 / 3-2009 pt.4.1	14.80 ± 0.2828 %, g/g

Table S3. Physico-chemical determination of apilarnil – Bulletin no. 947/12.12.2016 (ICDA Bucharest - Research and Development Institute for Beekeeping)

Physico-chemical determinations of apilarnil	Methods of analysis	Results
total proteins	LCPA // DFC-PS-10	7.5551 ± 0.5130 %, g/g
total fats	LCPA // DFC-PS-07 //Soxhlet	6.3916 ± 0.2788 %, g/g
ash	LCPA // DFC-PS-09	0.7508 ± 0.008 %, g/g
water	LCPA//DFC-PS-12<br>//oven drying method	71.4706 ± 0.8388 %, g/g

Determination of vitamin C in honey sample was performed using a titrimetric method according to Association of Official Analytical Chemists. Vitamin C in Juices and Vitamin Preparations. Official Method 967.21. In AOAC Official Methods of Analysis, 18th; Association of Official Analytical Chemists: Gaithersburg, MD, USA, 2005; pp 45.1.14.

Result: 1.54 ± 0.08 mg/100 g honey
